# Semaglutide and Its Potential Hepatoprotective Effects Against Acute Drug-Induced Liver Injury

**DOI:** 10.7759/cureus.107715

**Published:** 2026-04-25

**Authors:** Eftychia Charatsi, Evangelos Liberopoulos, Vasilios Pergialiotis, Konstantinos Kontzoglou, Stylianos Kykalos, Dimitrios Iliopoulos

**Affiliations:** 1 Second Department of Internal Medicine, Sismanogleio–Amalia Fleming General Hospital, Amalia Fleming Unit, Athens, GRC; 2 First Department of Propaedeutic and Internal Medicine, Laiko General Hospital, National and Kapodistrian University of Athens, Athens, GRC; 3 First Department of Obstetrics and Gynecology, Alexandra General Hospital, National and Kapodistrian University of Athens, Athens, GRC; 4 Second Department of Propaedeutic Surgery, Laiko General Hospital, School of Medicine, National and Kapodistrian University of Athens, Athens, GRC; 5 Second Department of Propaedeutic Surgery, Laiko General Hospital, National and Kapodistrian University of Athens, Athens, GRC; 6 Department of Cardiac Surgery, National and Kapodistrian University of Athens, Athens, GRC

**Keywords:** drug-induced liver injury, glp-1 receptor agonists, hepatoprotection, metabolic dysfunction-associated steatohepatitis, semaglutide

## Abstract

Drug-induced liver injury (DILI) is hepatic damage caused by medications, illegal drugs, herbal products, or dietary supplements and is related to inflammation, oxidative stress, and mitochondrial dysfunction. Manifestations can vary from isolated liver enzyme elevation to fulminant hepatic failure. As many well-established therapeutic options do not exist, it is crucial to explore new hepatoprotective agents. Semaglutide is a glucagon-like peptide-1 receptor agonist (GLP-1 RA), primarily used as an antidiabetic and anti-obesity agent, which manifests pleiotropic effects through activation of GLP-1 receptors. Recently, the Food and Drug Administration officially approved semaglutide for the treatment of metabolic dysfunction-associated steatohepatitis in adults with moderate-to-advanced fibrosis. The European Medicines Agency has issued a positive recommendation on the same indication. Proposed mechanisms for semaglutide’s liver beneficial effects include anti-inflammatory, antioxidant, and anti-apoptotic actions, as well as improved microcirculation, reduced lipotoxicity, and enhanced hepatic regeneration. Preclinical studies in models of toxic and ischemic liver injury demonstrate that GLP-1 RAs reduce hepatic damage. Semaglutide may offer therapeutic benefits in DILI, either as supportive treatment or as a stabilizing agent prior to transplantation. Still, further preclinical studies are required to provide the essential frame for clinical investigations in this setting.

## Introduction and background

Drug-induced liver injury (DILI) negatively impacts patients and health systems, as it is associated with 0.1%-3.0% of hospital admissions [1]. In prospective population-based studies, it is estimated that 14-19 per 100,000 citizens develop clinically significant DILI annually, requiring medical care [2].

DILI is related to the use of medications, not authorized drugs, herbal products, or dietary supplements [3]. Among the many substances implicated, certain drug classes are more frequently involved, such as anti-microbials, analgesics/anti-inflammatories, central nervous system agents, immunomodulatory agents, anti-neoplastic agents, and anti-viral agents [2]. It is one of the most common causes of acute liver injury (ALI); other etiologies include ischemia, autoimmunity, genetic disorders, viral infections, and alcohol use [4]. In the United States, DILI is responsible for 10%-15% of ALI cases [5].

The cornerstone of DILI treatment is the discontinuation of the responsible agent. Individuals with severe forms require hospital admission for supportive clinical treatment or eventual pharmaceutical interventions. Specific antidotes are effective in selected cases, such as N-acetylcysteine (NAC) in the case of liver damage by acetaminophen [5]. The administration of NAC plays a crucial role in the restoration of glutathione levels and the detoxification process, thereby minimizing liver cell injury. Liver transplantation is reserved for severe cases at risk of fulminant liver failure [6]. Corticosteroids represent a therapeutic approach for treating immune-mediated DILI, particularly in oncology patients, to manage hepatotoxicity related to immune checkpoint inhibitors [7].

Simultaneous use of certain medications (e.g., statins, beta-blockers, angiotensin-converting enzyme inhibitors, angiotensin II receptor blockers, and non-steroidal anti-inflammatory drugs) has shown a potential protective role in acetaminophen-associated liver events [2]. These findings suggest that drugs interact in complex ways, exerting biophysiological interplays that affect DILI outcomes [2]. Therefore, the use of medications with known hepatoprotective properties could favorably affect patients with DILI. Glucagon-like peptide-1 receptor agonists (GLP-1 RAs) represent a promising agent, as their beneficial role in chronic metabolic liver disease has been proven.

## Review

Pathophysiology of DILI

DILI occurs in susceptible individuals under the influence of genetic and environmental risk factors that are believed to modify drug metabolism and/or excretion [2]. Several factors influence the outcomes, including drug properties (dose, lipophilic profile, and pathway of hepatic metabolism) and individual characteristics (older age, sex, comorbidities, chronic liver disease, comedications, and prior alcohol use) [2]. Increased medication use among older adults and women may explain age- and sex-based differences reported in the incidence of DILI ​​​​[2].

Liver damage may vary in severity from asymptomatic elevation of liver enzymes to fulminant liver failure [3,5]. The liver function is deranged to a varying extent, leading to insufficient synthetic function of coagulation factors and albumin, failure of detoxification, and cholestasis [5].

In DILI, a cascade of cellular events occurs, involving multiple pathophysiological pathways induced by the parent drug or metabolically active compounds [8]. Major mechanisms include oxidative stress, cholestasis, and mitochondrial or endoplasmic dysfunction due to excessive generation of reactive oxygen species (ROS) [5,8,9]. Additionally, the responsible agent - directly or indirectly through hepatocyte injury - may cause immune-mediated damage by attacking liver antigens. This immune response involves activation of hepatic macrophages (Kupffer cells) and cytokine release of interleukin-1β (IL-1β), IL-6, and tumor necrosis factor (TNF-α) [10]. These pathways result in hepatocyte necrosis with the release of intracellular aminotransferases and hepatocyte apoptosis involving Jun N-terminal kinase (JNK) and caspase activation [11].

General and liver effects of GLP-1 RAs

GLP-1 RAs are a class of medications that act on the GLP-1 receptor, mimicking the actions of the endogenous incretin hormone GLP-1. GLP-1 RAs inhibit glucagon release and increase insulin secretion, induce satiety by delaying gastric emptying, and affect the central nervous system to decrease appetite [12]. Originally developed to treat type 2 diabetes, some GLP-1 RAs have been approved to treat obesity [13].

Semaglutide is a well-known GLP-1 RA, which has emerged as a promising therapy for a broad spectrum of metabolic, cardiovascular, renal, and hepatic disorders [14]. Semaglutide has pleiotropic effects, including glycemic and weight control, cardiovascular risk reduction, renal protection, and metabolic dysfunction-associated steatohepatitis (MASH) improvement [15].

Semaglutide effects on metabolic dysfunction-associated steatotic liver disease

Besides glycemic control, semaglutide exerts important functions in the liver. It reduces fat accumulation and improves metabolic dysfunction-associated steatotic liver disease (MASLD) and steatohepatitis (MASH), a condition associated with cirrhosis, hepatocellular carcinoma, and liver failure [16,17].

Evidence on the effects of semaglutide on MASH with fibrosis has emerged from the interim analysis of the ESSENCE study, an ongoing phase 3, multicentre, randomized, double-blind, placebo-controlled trial evaluating 800 patients at baseline and after 72 weeks. The primary endpoints were resolution of MASH with no worsening of liver fibrosis and the improvement of fibrosis with no progression of MASH after administration of semaglutide 2.4 mg once weekly or placebo in patients with MASH and stage 2-3 liver fibrosis. Resolution of MASH with no worsening of liver fibrosis was noted in 62.9% (n = 534) of patients in the semaglutide group compared with 34.3% (n = 266) in the placebo group (p < 0.001). Improvement of fibrosis with no progression of MASH occurred in 36.8% of patients in the semaglutide group versus 22.4% in the placebo group (p < 0.001) [18,19].

Preliminary data had been provided by a 72-week double-blind placebo-controlled trial including 320 patients with MASH and stage 1-3 liver fibrosis. Results showed higher rates of MASH resolution without worsening of liver fibrosis in the semaglutide group versus the placebo group (59% versus 17%, p < 0.001). No statistically significant difference in fibrosis improvement was observed [20]. In a 10-year retrospective study of 420 patients with diabetes and MASH receiving semaglutide, improved transaminases and MASH scores were attributed to semaglutide (p < 0.001) independently of weight loss [21]. In another large-scale cohort retrospective study conducted in more than 38,000 patients with MASLD, a slower progression to advanced liver disease and reduced cardiovascular events were reported with semaglutide treatment [16]. Also, in a prospective observational study of 70 diabetic patients with MASLD, semaglutide has been found to ameliorate liver steatosis and liver stiffness (p < 0.01) [22].

Based on the stated beneficial effects, the European Association for the Study of Diabetes (EASD) guidelines recommended incretin-based treatment for obese or diabetic patients with MASLD [23]. Recently, the Food and Drug Administration (FDA) [24] and the American Diabetes Association (ADA) recommended semaglutide for the treatment of MASH in adults with moderate-to-advanced fibrosis [25,26]. In January 2026, the European Medicines Agency (EMA) also gave a positive opinion for treating non-cirrhotic MASH with semaglutide (https://www.ema.europa.eu/en/medicines).

Semaglutide may improve liver steatosis and MASH primarily in indirect ways, as hepatocytes, Kupffer cells, and stellate cells do not express the GLP-1 receptor [27,28]. Mechanisms include weight loss and subsequent liver fat reduction, decreased dietary intake, improved insulin sensitivity, and a possible systemic anti-inflammatory effect [29]. Moreover, semaglutide may be associated with reduced de novo lipogenesis and increased fatty acid β-oxidation in the liver, which occurs through AMP-activated protein kinase (AMPK) and sirtuin-1 (SIRT-1) pathway activation [30], as well as ChREBP and SREBP-1c signaling downregulation [15,31].

Although it remains controversial, GLP-1 RAs may also act locally in the liver to reduce inflammation, as some hepatic endothelial and T cells appear to express GLP-1 receptors [32]. Their activation triggers signaling pathways such as cyclic AMP-protein kinase A (PKA), phosphatidylinositol 3-kinase-protein kinase B (PI3K-Akt), and AMPK, which are associated with anti-inflammatory effects [15]. Pro-inflammatory cytokines, such as IL-2, IL-4, and TNF-α, are also reduced, allowing for downscaling oxidative stress [15,33].

The hepatic safety of semaglutide is well documented in large clinical trials, including Semaglutide Unabated Sustainability in Treatment of Type 2 Diabetes (SUSTAIN 1-7), Semaglutide Treatment Effect in People with Obesity (STEP), and Semaglutide Effects on Cardiovascular Outcomes in People With Overweight or Obesity (SELECT), with only rare reported cases of hepatobiliary side effects [34]. As GLP-1 RAs can slow gallbladder emptying, bile stasis and gallstone formation may occur. Rapid weight loss increases cholesterol secretion and alters bile acid composition, favoring cholelithiasis and acute pancreatitis [35]. Still, GLP-1 RAs are safe for patients with MASH and compensated cirrhosis [36]. In a double-blind placebo-controlled phase 2 trial enrolling 70 cirrhotic overweight or obese patients, although semaglutide showed no improvement of liver fibrosis or MASH resolution after 48 weeks, it was well-tolerated, and no cirrhosis decompensation occurred [36].

Evidence of GLP-1 RA's hepatoprotective role in DILI

Animal models of DILI induced by drugs or toxins have demonstrated that GLP-1 RAs can attenuate hepatic damage [37-39]. In a study of atorvastatin-induced hepatotoxicity in rats, liraglutide improved liver function enzymes and histopathological findings by suppressing oxidative stress and inflammation [37]. Experimental models of CCL4-induced hepatoxicity in rats showed that liraglutide reduced acute hepatic injury [38,39]. Serum levels of ALT and AST were decreased, and the liver histopathological changes improved as a lower degree of necrosis was observed [38].

In preclinical models studying cases of ALI caused by ischemia, some GLP-1 RAs have shown improvement in liver histology and liver enzyme levels [40,41]. Liraglutide attenuated liver damage caused by hepatic ischemia-reperfusion injury [40], while exendin-4 (exenatide) ameliorated levels of liver function enzymes in diabetic rats subjected to renal ischemia-reperfusion [41].

Synthesis of the evidence

**Figure 1:**
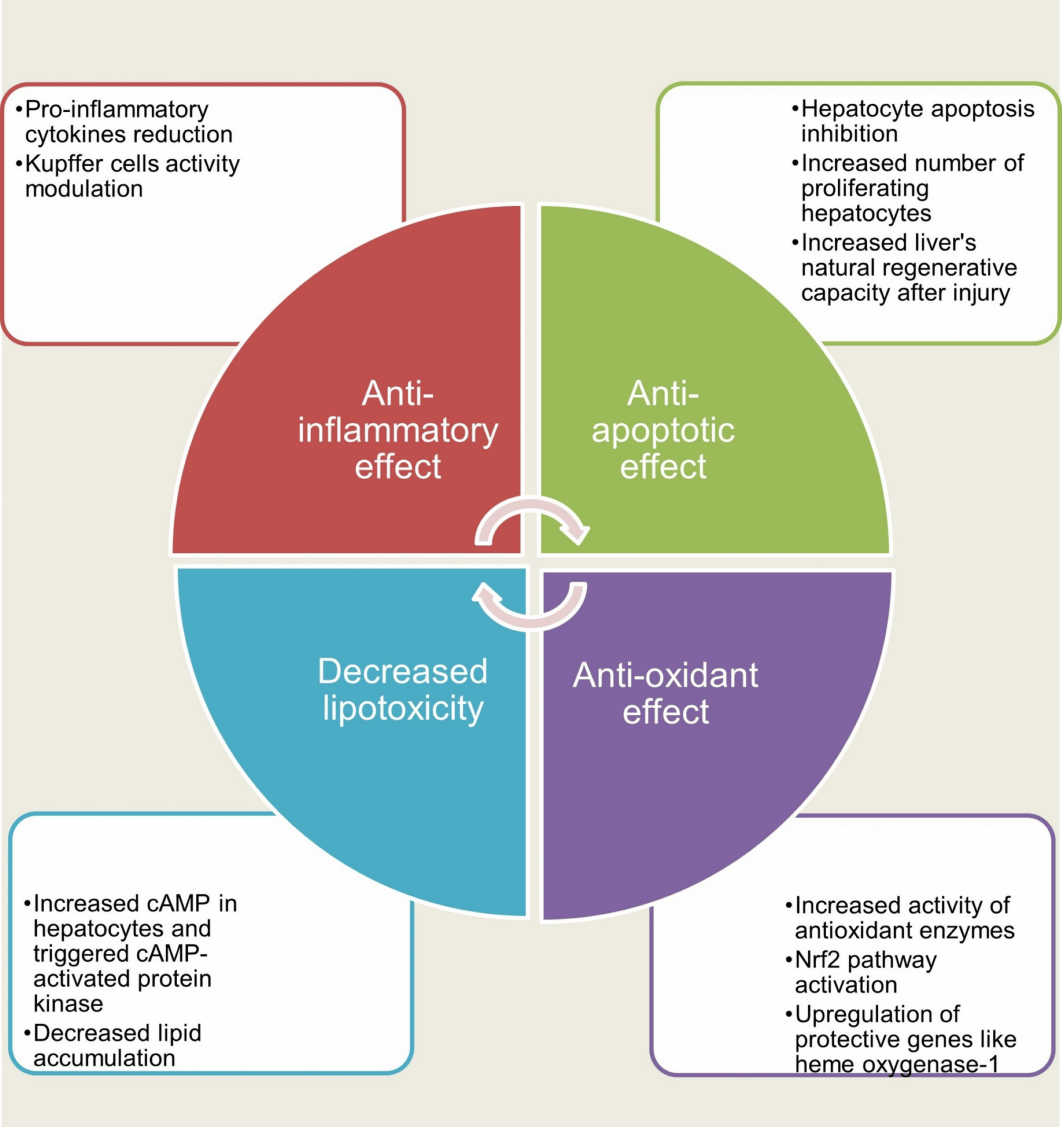
Potential hepatoprotective mechanisms of GLP-1 receptor agonists in drug-induced liver injury GLP-1 receptor agonists may exert hepatoprotective effects through multiple complementary mechanisms, including antioxidant activity, suppression of inflammatory signaling, inhibition of hepatocyte apoptosis, and reduction of lipotoxicity. These effects involve activation of antioxidant pathways (e.g., Nrf2/HO-1), modulation of Kupffer cell-mediated inflammatory responses, enhancement of hepatocyte survival and regenerative capacity, and AMPK-mediated regulation of hepatic lipid metabolism. Collectively, these mechanisms may improve hepatic microcirculation and support liver recovery after toxic injury. Current evidence is largely derived from experimental studies [37-43]. This figure was created using Microsoft Word’s SmartArt (Microsoft Corp., Redmond, WA). AMPK: AMP-activated protein kinase; Nrf2: Nuclear factor erythroid 2-related factor 2; GLP-1: Glucagon-like peptide-1.

GLP-1 RAs have been increasingly recognized for their potential hepatoprotective effects through multiple complementary mechanisms, including antioxidant, anti-inflammatory, anti-apoptotic, and metabolic actions that reduce lipotoxic stress and support hepatic recovery after injury. Experimental and translational evidence suggests that these protective effects are mediated through both direct signaling within hepatocytes and indirect modulation of the hepatic inflammatory and metabolic microenvironment [32,33] (Figure 1).

Oxidative stress is a central driver of hepatocyte injury in toxic and metabolic liver diseases, including DILI. GLP-1 RAs appear to enhance endogenous antioxidant defenses by increasing the activity of key antioxidant enzymes such as superoxide dismutase (SOD), glutathione peroxidase (GPx), and catalase (CAT), thereby limiting the accumulation of ROS within hepatic cells [42]. In addition, GLP-1 receptor activation has been shown to stimulate the nuclear factor erythroid-2 related factor-2 (Nrf2) signaling pathway, a major regulator of cellular defense against oxidative stress [43]. Activation of Nrf2 leads to transcriptional upregulation of several cytoprotective genes, including heme oxygenase-1 (HO-1) and other detoxifying enzymes that protect hepatocytes from oxidative injury [43]. Experimental studies have further demonstrated that GLP-1 analogs such as liraglutide can attenuate toxin-induced hepatotoxicity by restoring GLP-1 receptor expression and activating Nrf2-mediated antioxidant pathways, thereby reducing oxidative damage and improving cellular resilience [37].

In addition to antioxidant effects, GLP-1 RAs exert important anti-inflammatory actions within the liver. Chronic hepatic inflammation is a key component of both metabolic and toxic liver injury, contributing to hepatocyte damage and disease progression. GLP-1-based therapies have been shown to reduce circulating levels of pro-inflammatory cytokines and modulate immune signaling pathways involved in hepatic inflammation [33]. At the level of the liver, GLP-1 signaling can influence the activity and phenotype of Kupffer cells, the resident hepatic macrophages that orchestrate inflammatory responses. Experimental models have demonstrated that liraglutide promotes polarization of Kupffer cells toward an anti-inflammatory M2 phenotype through activation of the cAMP-PKA-STAT3 signaling pathway, resulting in reduced inflammatory stress and improved hepatic homeostasis [44]. Similar immunomodulatory effects have also been observed in models of hepatic ischemia-reperfusion injury, where GLP-1 analogs attenuated liver damage by altering macrophage responses and limiting inflammatory cascades [40].

GLP-1 RAs may also protect hepatocytes by limiting apoptosis and enhancing regenerative processes following liver injury. Apoptotic cell death is a prominent mechanism in toxic and inflammatory liver damage, and GLP-1 signaling has been shown to suppress hepatocyte apoptosis in several experimental models. Preclinical studies indicate that GLP-1 analogs can reduce hepatocyte apoptosis following toxic insults and ALI, thereby preserving hepatic cellular integrity [37,38]. Furthermore, some investigations have suggested that pretreatment with GLP-1 RAs increases the number of proliferating hepatocytes and enhances the liver’s intrinsic regenerative capacity after injury. In murine models of chemically induced liver damage, liraglutide administration resulted in improved histological recovery and increased markers of hepatocyte proliferation during the regenerative phase [39].

Another important hepatoprotective mechanism of GLP-1 RAs involves the reduction of hepatic lipotoxicity. Excessive lipid accumulation within hepatocytes contributes to oxidative stress, mitochondrial dysfunction, and inflammatory signaling. GLP-1 receptor activation has been shown to increase intracellular cyclic adenosine monophosphate (cAMP) levels in hepatocytes, leading to activation of AMP-activated protein kinase (AMPK), a key metabolic regulator that suppresses de novo lipogenesis and promotes fatty acid oxidation [30]. Through this pathway, GLP-1 RAs reduce hepatic lipid accumulation and improve metabolic homeostasis within liver tissue. Evidence also suggests that GLP-1 receptors are expressed on human hepatocytes and that GLP-1 signaling can directly modulate elements of the insulin signaling pathway, thereby reducing hepatic steatosis and improving cellular metabolic balance [32]. Clinical and translational studies evaluating GLP-1 RAs such as semaglutide have similarly demonstrated reductions in liver fat content in patients with metabolic-associated steatotic liver disease, supporting the metabolic component of these hepatoprotective effects [31].

Taken together, these findings suggest that the hepatoprotective effects of GLP-1 receptor agonists arise from a multifactorial interplay of antioxidant, anti-inflammatory, anti-apoptotic, and metabolic mechanisms that collectively mitigate hepatocellular injury and promote tissue recovery (Table 1). Importantly, many of these effects appear to involve direct cellular actions within hepatic tissue rather than solely systemic metabolic improvements. It should be noted that the hepatoprotective function of GLP-1 RAs may also extend to acute hepatotoxicity, as experimental models indicate that GLP-1 receptor activation can enhance hepatocellular resilience to toxic insults, attenuate biochemical markers of liver injury, and improve histological outcomes when administered before or during the early phase of hepatic damage. These protective effects are thought to result from stabilization of mitochondrial function, preservation of intracellular redox balance, and modulation of hepatic immune responses, which together limit the cascade of oxidative injury and inflammatory amplification that typically follows toxic exposure. Furthermore, GLP-1 signaling has been associated with improved sinusoidal microcirculation and maintenance of hepatocyte metabolic homeostasis during acute stress conditions, factors that may facilitate more efficient recovery of liver architecture and function.

**Table 1:** Proposed hepatoprotective mechanisms of GLP-1 receptor agonists and their potential relevance to semaglutide in DILI The table summarizes established class effects of GLP-1 receptor agonists, current evidence specific to semaglutide, and the presumed implications for acute hepatotoxicity. Most mechanistic data are derived from experimental models, while semaglutide evidence in DILI remains limited, supporting the need for further preclinical and clinical investigation [32,33,37-41]. GLP-1 RA: Glucagon-like peptide-1 receptor agonist; DILI: Drug-induced liver injury; SOD: Superoxide dismutase; GPx: Glutathione peroxidase; CAT: Catalase; ROS: Reactive oxygen species; Nrf2: Nuclear factor erythroid-2-related factor 2; AMPK: AMP-activated protein kinase; MASLD: Metabolic dysfunction-associated steatotic liver disease; MASH: Metabolic dysfunction-associated steatohepatitis.

Mechanistic domain	Established evidence for GLP-1 RAs	Evidence specific to semaglutide	Presumed relevance in DILI
Oxidative stress regulation	Activation of antioxidant enzymes (SOD, GPx, CAT) and Nrf2-mediated cytoprotective pathways; reduction of ROS-mediated hepatocyte injury.	Direct evidence of acute hepatotoxicity is lacking; indirect support from improved hepatic metabolic profile in steatotic liver disease.	May protect hepatocytes from oxidative damage during toxic exposure and early cellular stress.
Anti-inflammatory activity	Reduction of pro-inflammatory cytokines and modulation of Kupffer cell/macrophage activity; promotion of anti-inflammatory M2 polarization.	Systemic anti-inflammatory effects described in metabolic disease; direct DILI data unavailable.	May attenuate inflammatory processes that contribute to hepatocellular injury progression.
Anti-apoptotic and cytoprotective effects	Experimental models show reduced hepatocyte apoptosis and preservation of cellular integrity after toxic injury.	No direct semaglutide data in acute toxic liver injury.	Could limit progression from reversible cellular stress to irreversible hepatocyte death.
Metabolic stabilization and reduced lipotoxicity	Activation of AMPK signaling and suppression of hepatic lipogenesis leading to decreased intracellular lipid accumulation.	Semaglutide reduces liver fat content and improves histological markers in MASLD/MASH.	Improved metabolic environment may increase hepatic resilience to hepatotoxic drugs, particularly in metabolically vulnerable patients.
Hepatic regeneration and repair	Preclinical studies suggest increased hepatocyte proliferation and enhanced regenerative response after injury.	No direct semaglutide evidence in acute injury models.	May support restoration of liver architecture following toxic insult.
Microcirculatory and ischemia-related protection	GLP-1 analogs attenuate hepatic ischemia-reperfusion injury in experimental models.	No semaglutide-specific data are currently available.	Potential stabilization of sinusoidal microcirculation during severe hepatic injury.
Overall translational implication	Class-level hepatoprotective effects are demonstrated mainly in experimental toxic liver injury models.	Strong clinical evidence for metabolic liver disease, but not yet for DILI.	Semaglutide may represent a candidate supportive therapy in DILI, pending preclinical and clinical validation.

Although the current evidence base is largely derived from experimental and preclinical studies, these observations highlight the potential of GLP-1 RAs as supportive therapeutic agents in conditions characterized by hepatocellular injury, including DILI. However, it should be noted that due to this fact, the current level of evidence and strength of recommendation across the mechanistic modes of action of GLP-1 RAs remains limited and therefore should not be considered in routine practice (Table 2).

**Table 2:** Levels of evidence and grade of recommendation for GLP-1 RAs Levels of evidence and grades of recommendation are derived by the author and reflect the consistency, directness, and translational relevance of currently available experimental and clinical data [15,33,37-41]. Nrf2: Nuclear factor erythroid-2-related factor 2; MASLD: Metabolic dysfunction-associated steatotic liver disease; MASH: Metabolic dysfunction-associated steatohepatitis; AMPK: AMP-activated protein kinase; DILI: Drug-induced liver injury; GLP-1: Glucagon-like peptide-1.

Mechanistic domain	Level of evidence	Grade of recommendation	Basis for grading
Oxidative stress regulation	Moderate	B	Multiple experimental studies show activation of antioxidant enzymes and Nrf2-mediated cytoprotective pathways. Evidence is consistent but largely preclinical.
Anti-inflammatory activity	Moderate	B	Experimental data demonstrate reduction of pro-inflammatory cytokines and modulation of Kupffer cell activity; biologically consistent across models of liver injury.
Metabolic stabilization/reduced lipotoxicity	Moderate	B	Strong mechanistic evidence for AMPK activation and reduced hepatic lipid accumulation; indirect clinical support from MASLD/MASH studies.
Anti-apoptotic/cytoprotective effects	Low	C	A limited number of experimental studies suggest reduced hepatocyte apoptosis after toxic injury.
Hepatic regeneration and repair	Low	C	Preliminary animal data suggest increased hepatocyte proliferation and improved recovery following injury.
Microcirculatory/ischemia-related protection	Low	C	Evidence mainly derived from ischemia-reperfusion models rather than classical DILI models.
Direct hepatic cellular signaling	Low	C	Some evidence supports the presence of GLP-1 receptor in hepatocytes and direct metabolic signaling, but findings remain debated.
Semaglutide-specific role in acute DILI	Very low	D	Current evidence is indirect and extrapolated from class effects and metabolic liver disease studies.

## Conclusions

DILI remains a major cause of both acute and chronic hepatic dysfunction and represents one of the leading causes of acute liver failure in many countries. Severe cases may progress rapidly to hepatic failure requiring urgent liver transplantation, while therapeutic options remain largely supportive. Given these limitations, investigation of pharmacologic agents with potential hepatoprotective properties, including GLP-1 RAs, represents an important area of translational research. Considering the encouraging hepatoprotective effects across several models of toxic liver injury, it could be assumed that there is a mechanistic basis for exploring whether similar benefits may be observed with semaglutide, which has a favorable pharmacokinetic profile and well-established safety record in metabolic disease. The pleiotropic cellular actions of GLP-1 receptor signaling, including regulation of oxidative stress pathways, modulation of hepatic immune responses, improvement of intracellular metabolic balance, and enhancement of hepatocyte survival, suggest that this drug class could modify key pathophysiological processes involved in toxic liver injury. Beyond the mechanistic rationale, several translational scenarios can be envisioned in which GLP-1 RAs might provide clinical benefit. First, these agents could potentially function as supportive hepatoprotective therapies during the early phases of DILI, aiming to stabilize hepatocellular function while the offending agent is withdrawn and endogenous repair mechanisms are activated. Second, in severe cases of ALI or impending liver failure, pharmacologic agents that limit ongoing hepatocyte loss and preserve residual liver function may help bridge patients to recovery or transplantation, thereby improving clinical outcomes. Third, GLP-1 RAs might have value in patients with underlying metabolic liver disease, in whom hepatic steatosis and metabolic dysfunction may increase susceptibility to drug-induced hepatotoxicity. Another important translational implication relates to the possibility that GLP-1 RAs may influence hepatic regenerative processes. Experimental findings suggest that activation of GLP-1 receptor signaling can promote hepatocyte proliferation and improve the regenerative response following injury. If confirmed in human studies, this effect could have relevance not only for DILI but also for broader clinical contexts involving acute hepatic damage.

Despite these promising theoretical considerations, several important knowledge gaps remain. The majority of available evidence derives from animal and cellular models, and the extent to which these findings translate to human disease is currently uncertain. Future preclinical investigations should therefore focus on clarifying the specific molecular pathways involved in GLP-1-mediated hepatoprotection, determining optimal dosing and timing of administration in the setting of toxic injury, and evaluating potential interactions with commonly implicated hepatotoxic medications. Clinical research will also be required to define the safety and therapeutic profile of semaglutide in patients with ALI. Although GLP-1 RAs are generally well-tolerated, rare cases of liver injury have been reported, and careful evaluation of the risk-benefit balance will be necessary in this vulnerable population. Prospective clinical studies, including observational cohorts and early-phase trials, may help determine whether GLP-1-based therapies can meaningfully modify disease course in DILI. Provided that a hepatoprotective function is established, semaglutide could emerge as a novel adjunctive therapeutic option in DILI, either as supportive therapy during the acute phase of injury or as a stabilizing intervention in patients with severe hepatic dysfunction awaiting transplantation. Continued integration of experimental findings with carefully designed clinical studies will be essential to determine whether the promising biological properties of GLP-1 receptor agonists can translate into meaningful therapeutic benefit in DILI.
